# Supplementary material for: Prolactin Regulates Ovine Ovarian Granulosa Cell Apoptosis by Affecting the Expression of MAPK12 Gene
Source: Int J Mol Sci. 2023 Jun 17;24(12):10269. doi: 10.3390/ijms241210269 (PMC10298867; doi:10.3390/ijms241210269)
Supplement: Supplementary file 1 [file ijms-24-10269-s001.zip › Supp Table S1.pdf]

Supplementary Table S1. Ingredients and chemical composition of the diet for the steers as rumen fluid donor (% DM)

Items	Contents
Peanut seedling	14.5
Corn Silage	53.2
Corn	9.1
Bran	6.9
Soybean meal	8.5
Com DDGS	4.8
Stone powder	0.9
NaCl	0.6
NaHCO3	1.0
Premix	0.5
Nutrients	
CP	13.25
NDF	24.62
ADF	23.61
EE	7.27
Ca	1.30
P	0.7
ME (MJ/kg)	9.68

Note: The premix provided the following per kg of the diet: VA 11500 IU, VD25000 IU, VE 600 mg, nicotinic acid 300 mg, Zn (as zinc sulfate) 2.8 g, Cu (as copper sulfate) 0.5 g, Se (as sodium selenite) 6.5 mg, Fe (as ferrous sulfate) 1.0 g, Mn (as manganese suIfate) 110 mg.

CP = crude protein; ADF = acid detergent fiber; NDF = neutral detergent fiber.
