# Supplementary material for: Prolactin Regulates Ovine Ovarian Granulosa Cell Apoptosis by Affecting the Expression of MAPK12 Gene
Source: Int J Mol Sci. 2023 Jun 17;24(12):10269. doi: 10.3390/ijms241210269 (PMC10298867; doi:10.3390/ijms241210269)
Supplement: Supplementary file 1 [file ijms-24-10269-s001.zip › Supp Table S2.pdf]

Supplementary Table S2. Experimental design

| | | |
|---|---|---|
| | | Experimental design |
| Step 1 | Correlation between PRL and follicles counts | **Group A**, sponge withdrawl (day 8th) |
| | | **Group B**, sponge withdrawl (day 11th) |
| | | **Group C**, FSH (day 7th) + sponge withdrawl (day 8th) |
| | | **Group D**, FSH (day 10th) + sponge withdrawl (day 11th) |
| Step 2 | Knockdown of *L-PRLR* and *S-PRLR* by CRISPR/Cas9 | **P**, GCs with HPC (500 ng/mL PRL) |
| | | **P-458** (Negative control group), GCs with HPC were transfected by empty plasmid pX458 |
| | | **P-sg-L**, GCs with HPC were transfected by recombinant plasmid of knock down *L-PRLR* |
| | | **P-sg-SL**, GCs with HPC were transfected by recombinant plasmid of knock down both *L-PRLR* and *S-PRLR* |
| | Overexpression of *L-PRLR* and *S-PRLR* | **P**, GCs with HPC |
| | | **P-10** (Negative control group), HPC were infected by lentiviruses with empty vectors pGWLV10-new of overexpression |
| | | **P-10-L**, GCs with HPC were infected by lentiviruses carrying overexpressed sequences of *L-PRLR* |
| | | **P-10-S**, GCs with HPC were infected by lentiviruses carrying overexpressed sequences of *S-PRLR* |
| Step 3 | Interference of *MAPK12* | **P**, GCs with HPC |
| | | **P-G** (Negative control group), GCs with HPC were infected by lentiviruses with empty vectors pGWLV33 of interference |
| | | **P-SH**, GCs with HPC were infected by lentiviruses carrying interference sequence of *MAPK12* |
| | Overexpression of *MAPK12* | **P**, GCs with HPC |
| | | **P-10**, (Negative control group), HPC were infected by lentiviruses with empty vectors pGWLV10-new of overexpression |
| | | **P-10-M**, GCs with HPC were infected by lentiviruses carrying overexpressed sequences of *MAPK12* |
