# Supplementary material for: Prolactin Regulates Ovine Ovarian Granulosa Cell Apoptosis by Affecting the Expression of MAPK12 Gene
Source: Int J Mol Sci. 2023 Jun 17;24(12):10269. doi: 10.3390/ijms241210269 (PMC10298867; doi:10.3390/ijms241210269)
Supplement: Supplementary file 1 [file ijms-24-10269-s001.zip › Supplemental File S1.pdf]

***L-PRLR*:**GCTAGCGCCACCATGAAGGAAAATGCAGCATCCAGAGTGCTTTTCAT

TCTGCTACTTTTTCTCTTCGCCAGCCTTCTGAATGGACAGTCACCTCCTGAAAA

ACCCAAGCTTATTAAATGTCGGTCTCCTGGAAAGGAAACGTTCACCTGCTGGT

GGGAGCCCGGGGCAGATGGAGGACTTCCTACCAATTACACACTGACTTACCGC

AAGGAAGGAGAAACACTCATCCATGAATGTCCAGACTACAAAACCGGGGGCC

CCAACTCCTGCTACTTTAGCAAGAAGTACACCTCCATATGGAAGATGTATGTCA

TCACAGTAAGCGCCATCAACCAGATGGGAATCAGTTCCTCAGATCCACTTTAT

GTGGACGTGACTTACATAGTTGAACCAGAGCCTCCTGTGAACCTGACTTTGGA

ATTAAAACATCCAGAAGATAGAAAACCATATCTATGGATAAAATGGTCTCCACC

CACCCTGACTGATGTAAAATCTGGTTGGTTCAGTATCCAGTACGAAATTCGATT

AAAACCTGAGAAAGCAACTGACTGGGAGACTCATTTTGCTCCAAAGCTGACT

CAGCTTAAGATTTTCAACTTATATCCAGGACAGAAATACCTTGTGCAGATTCGA

TGCAAGCCAGACCATGGATACTGGAGTGAGTGGAGCCCAGAGAGCTTCATCC

AGATACCTAATGACTTCCCAGTGAAGGATACAAGCATGTGGATCTTTGTGGGC

GTCCTTTCTGCTGTCATCTGTTTGATTATGGTCTGGGCAGTGGCTTTGAAGGGC

TATAGCATGGTGACCTGCATCCTCCCACCAGTTCCAGGGCCAAAAATAAAAGG

ATTTGATATTCATCTGCTGGAGAAGGGCAAGTCCGAAGAACTTCTGAGAGCTC

TGGAAAGCCAAGACTTCCTTCCCACTTCTGACTGCGAGGATTTGCTGATGGAA

TTCATAGAGGTAGATGACAGTGAGGACCAACACCTGATGCCACACCCCTCCAA

AGAACACATGGAGCAAGGCGTGAAGCCCATGCACCTGGATCCTGACACTGAC

TCTGGCCGGGGCAGCTGTGACAGCCCTTCACTCTTGTCTGAAAAGTGTGATGA

ACCTCAGGCCTATCCCTCCAAGTTCCACATTCCAGAGGGCCCTGAGAAGCTGG

AGGATCCCGAAACAAATCATACATGTCTCCAGGCCCCTCAGAGCACAAGTGGG

GAAGGCAAAATCCCCTATTTTCTGGCCAACGGACCCAAATCTTCCACATGGCC
TTTCCCGCAGCCCCCCAGCCTGTACAGCCCCAGATATTCTTACCACAACATTGC
TGACGTGTGTGAGCTGGCCCTGGGCATGGCAGGCACCACAGCCACTCTGCTG
GACCAAACAGACCAACATGCCTTTAAACCCTCAAAAACCATTGAGACTGGCG
GGGAAGGAAAGGCAGCCAAACAGAGCGAGTCAGAAGGCTACAGTTCCGAGC
CTGACCAAGACATGGCATGGCCACTGCTCCAAGACAAAACCCCCTTGTTCTCT
GCTAAACCCTTGGAATATGTGGAGATCCACAAGGTCAGCCAAGATGGAGTGCT
AGCTCTGTTCCCAAAACAAAATGAGAAGGTTGACGCCCCTGAAACCAGCAAG
GAGTACTCGAAGGTGTCTCGGGTGACGGATAGCAACATCCTGGTGTTGATACC
GGATCTGCAAGCGCAAAACCTGACTCTGTTAGAAGAATCAGCCAAGAAGGCC
CCGCCAGCCCTGCCATAGTCTAGA

***S-PRLR*:**GCTAGCGCCACCATGAAGGAAAATGCAGCATCCAGAGTGCTTTTCAT
TCTGCTACTTTTTCTCTTCGCCAGCCTTCTGAATGGACAGTCACCTCCTGAAAA
ACCCAAGCTTATTAAATGTCGGTCTCCTGGAAAGGAAACGTTCACCTGCTGGT
GGGAGCCCGGGGCAGATGGAGGACTTCCTACCAATTACACACTGACTTACCGC
AAGGAAGGAGAAACACTCATCCATGAATGTCCAGACTACAAAACCGGGGGCC
CCAACTCCTGCTACTTTAGCAAGAAGTACACCTCCATATGGAAGATGTATGTCA
TCACAGTAAGCGCCATCAACCAGATGGGAATCAGTTCCTCAGATCCACTTTAT
GTGGACGTGACTTACATAGTTGAACCAGAGCCTCCTGTGAACCTGACTTTGGA
ATTAAAACATCCAGAAGATAGAAAACCATATCTATGGATAAAATGGTCTCCACC
CACCCTGACTGATGTAAAATCTGGTTGGTTCAGTATCCAGTACGAAATTCGATT
AAAACCTGAGAAAGCAACTGACTGGGAGACTCATTTTGCTCCAAAGCTGACT

CAGCTTAAGATTTTCAACTTATATCCAGGACAGAAATACCTTGTGCAGATTCGA
TGCAAGCCAGACCATGGATACTGGAGTGAGTGGAGCCCAGAGAGCTTCATCC
AGATACCTAATGACTTCCCAGTGAAGGATACAAGCATGTGGATCTTTGTGGGC
GTCCTTTCTGCTGTCATCTGTTTGATTATGGTCTGGGCAGTGGCTTTGAAGGGC
TATAGCATGGTGACCTGCATCCTCCCACCAGTTCCAGGGCCAAAAATAAAAGG
ATTTGATATTCATCTGCTGGAGATATCACAGCCTTCTCGCCTTGTGTCTGTGTTT
TAATCTAGA

MAPK12:GCTAGCGCCACCATGAGCTCCCCGTCGCCCGCCCGCAAGGGCTTTTA
TCGCCAGGAGGTGACCAAGACGGCCTGGGAGGTGCGCGTCGTGTACCAGGAT
CTGCAGCCCGTGGGCTCCGGCGCCTACGGCGCCGTGTGCTCGGCGGTGGACA
GCCGCACGGGCGCCAAGGTGGCCATCAAGAAGCTGTACCGGCCCTTCCAGTC
CGAGCTGTTCGCCAAGCGCGCCTACCGCGAGCTGCGCCTGCTGAAGCACATG
CGCCACGAGAACGTGATTGGGCTGCTGGACGTGTTCACGCCCGATGAGACAC
TGGATGACTTCACGGACTTCTACCTGGTGATGCCGTTCATGGGCACCGACCTG
GGAAAGCTCATGAAGCACGAGAAGCTGAGCGAAGACCGGGTGCAGTTCCTC
GTCTACCAGACGCTTAAGGGGCTGAAGTACATCCACGCTGCTGGCGTCATCCA
TAGGGACTTGAAGCCCAGCAACCTGGCTGTGAACGAGGACTGTGAGCTGAAG
ATTCTGGACTTCGGCCTGGCCCGGCAGGCAGACAGCGAGATGACCGGCTACG
TGGTGACCCGGTGGTACCGCGCGCCCGAGGTCATCTTGAACTGGATGCGCTAC
ACGCAGACGGTGGACATCTGGTCCGTGGGCTGCATCATGGCTGAGATGATCAC
AGGGAAGACGCTCTTCAAAGGCAACGACCACCTGGACCAGCTGAAGGAGAT
CATGAAGGTGACAGGGACGCCTCCCGCGGAGTTTGTGCAGAGGCTGCAAAGT

GATGAGGCCAAGAACTACATGAAGGGCCTCCCTGAGCTGGAGAAGAAGGATT

TTGCCTCCGTCCTGACCAACGCGAGCCCCCTGGCCGTGAGCCTCCTGGAGAA

AATGCTGGTGCTGGACGCAGAGCAGCGGGTGACGGCGGCCGAGGCACTGGC

CCATCCCTACTTCGAGTCACTGCACGACACGGAGGACGAGCCCCAGGCCCAG

AAGTACGATGAGTCCTTTGACGACGTGGACCGCACGCTGGATGAGTGGAAGC

GTGTCACATATAAAGAGGTGCTCAGCTTCAAGCCTCCCCGGCAGCTGGGGGCC

AAGGCCTCCAAGGAGACAGCCTTGTGATCTAGA
