# Supplementary material for: Prolactin Regulates Ovine Ovarian Granulosa Cell Apoptosis by Affecting the Expression of MAPK12 Gene
Source: Int J Mol Sci. 2023 Jun 17;24(12):10269. doi: 10.3390/ijms241210269 (PMC10298867; doi:10.3390/ijms241210269)
Supplement: Supplementary file 1 [file Figure S1.pdf › Figure S1.pdf]

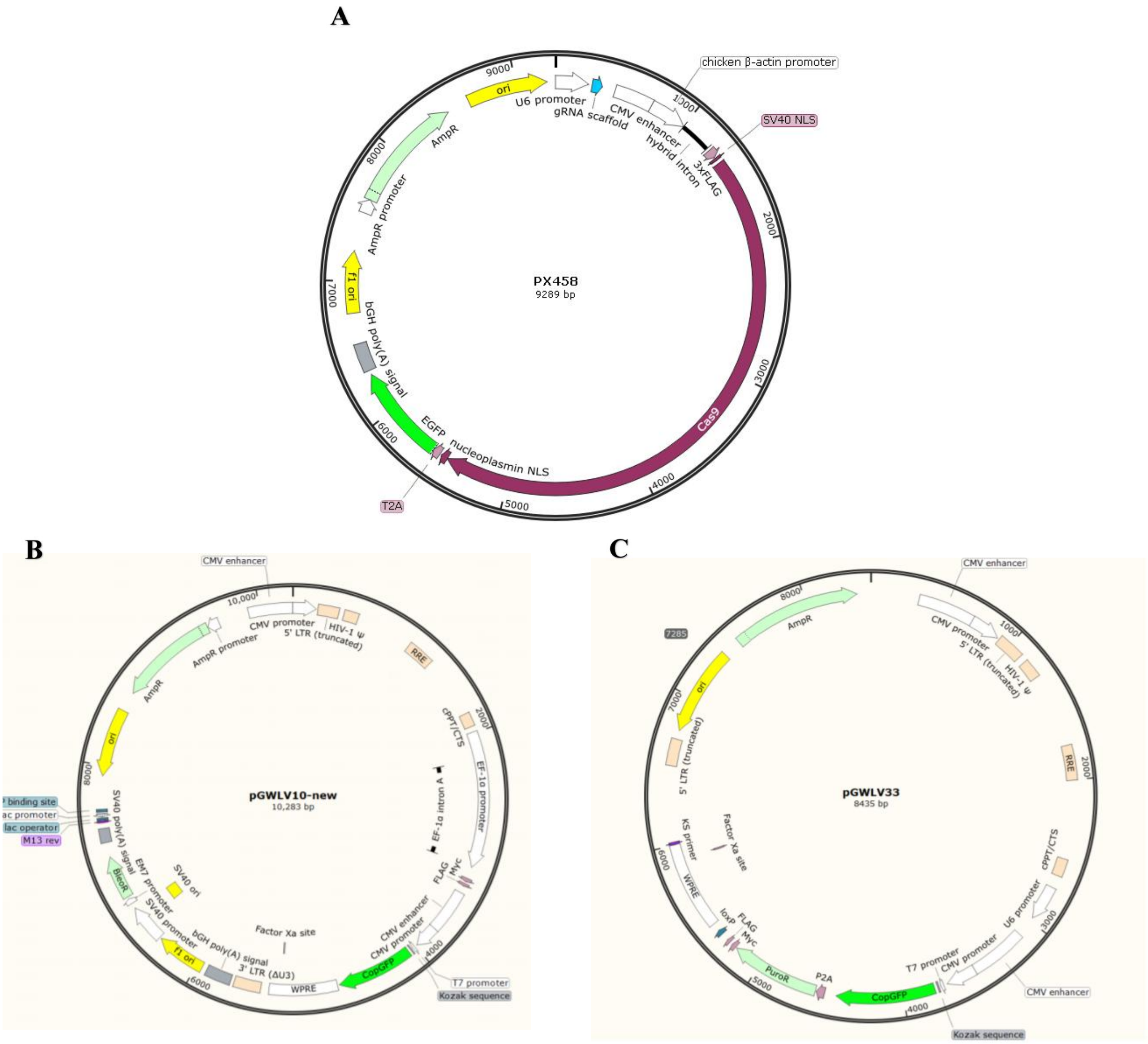

Figure S1. The empty plasmid. A: Plasmid PX458; B: Plasmid pGWLV10-new; C: Plasmid pGWLV33.
